# Photocurable High-Energy Polymer-Based Materials for 3D Printing

**DOI:** 10.3390/polym15214252

**Published:** 2023-10-28

**Authors:** Dmitrii Tkachev, Yana Dubkova, Alexander Zhukov, Yanis Verkhoshanskiy, Alexander Vorozhtsov, Ilya Zhukov

**Affiliations:** Laboratory of Metallurgy Nanotechnologies, National Research Tomsk State University, Lenin Avenue, 36, 634050 Tomsk, Russia; d.tkachev11@gmail.com (D.T.); ya.a.dubkova@yandex.ru (Y.D.); verkhoshanskiy@yandex.ru (Y.V.); abv195@mail.ru (A.V.); gofra930@gmail.com (I.Z.)

**Keywords:** additive manufacturing, stereolithography, structure, burning rate, photopolymer, high-energy materials

## Abstract

Digital light processing (DLP) or stereolithography is the most promising method of additive manufacturing (3D printing) of products based on high-energy materials due to, first of all, the absence of a high-temperature impact on the material. This paper presents research results of an ultraviolet (UV)-cured urethane methacrylate polymer containing 70 wt.% of high-energy solid powder based on ammonium salts, which is intended for digital light processing. Polymerization of the initial slurry is studied herein. It is shown that the addition of coarse powder transparency for the UV radiation to resin increases its curing depth. The thickness of the layer, which can polymerize, varies from 600 µm to 2 mm when the light power density ranges from 20 to 400 mJ/cm^2^, respectively. In DLP-based 3D printing, the obtained material density is 92% of the full density, while the compressive strength is 29 ± 3 MPa, and the ultimate tensile strength is 13 ± 1.3 MPa. The thermogravimetric analysis shows the decrease in the thermal decomposition temperature of UV-cured resin with high-energy additives compared to the thermal decomposition temperatures of the initial components separately. Thermal decomposition is accompanied by intensive heat generation. The burning rate of obtained samples grows from 0.74 to 3.68 mm/s, respectively, at the pressure growth from 0.1 to 4 MPa. Based on the results, it can be concluded that DLP-based 3D printing with the proposed UV photocurable resin is rather promising for the fabrication of multicomponent high-energy systems and complex profile parts produced therefrom.

## 1. Introduction

It is known that geometric parameters of products based on high-energy materials (HEMs) exert a direct effect on their functional properties (gas generation rate and volume, process completeness) [1,2]. Additive manufacturing (AM) is the up-to-date method of fabricating products with complex geometry using various materials. Owing to a high labor intensity conditioned by the risk of fire and explosion, additive manufacturing of HEM-based products is currently being intensively developed, including investigations of the development of methods, process conditions, and compositions for 3D printing.

HEM-based compositions have an elastic polymer matrix, allowing the creation of the required geometry and physical and mechanical characteristics. In this polymer matrix, solid powder distribution is uniform, and its composition is characterized by a higher energy release during oxidation and decomposition reactions. In the conventional AM technique, liquid materials are mostly used to obtain the desired geometry for HEM-based products. These materials are butadiene rubbers, which polymerize after the addition of curing agents during chemical reactions. The commonly used polymers could be either inert, such as HTPB, or active, such as azide polymers [3]. Researchers involved in the development of methods and compositions for HEM 3D printing, thus, pay particular attention to the AM adaptation to polymer-based materials and the creation of polymer compositions working as the matrix for the HEM addition during product fabrication. The literature [4,5,6] shows examples both of the adaptation of typical HEM composition for 3D printing fabrication and of the solid HEM addition to typical polymers used in 3D printing such as polylactide (PLA)- and acrylonitrile butadiene styrene (ABS)-based polymers for fused deposition modeling (FDM) 3D printing. For example, a group of Indian researchers has shown the possibility of the extrusion-based 3D printing of the composition based on AP with HTPB as a binder [5]. The authors of [6] have shown the possibility of adapting the PLA in order to create FDM 3D-printable HEM, also based on AP. However, the main problem in using the 3D printing approaches based on the extrusion, especially for HEM 3D printing, is the material heating up to 200 °C, which is rather laborious for the production of HEMs, which are resistant to such temperatures. Robocasting is an alternative approach to HEM 3D printing based on material extrusion [7]. In this approach, the extruded material solidifies after a rapid vaporization of the liquid binder, either exposed to UV radiation or due to the plasticity of the initial material. In [8,9,10], it is shown that high-energy slurries with a high content of powdered material can be used in robocasting. The main weakness of this 3D printing approach is low fidelity owing to the nozzle diameter and cure rate of the material. In addition to robocasting, stereolithography (SLA) or digital light processing (DLP) is a rather promising additive manufacturing method, which uses a focused UV laser onto a vat of photopolymer resin. The light can be emitted by a laser or light-emitting diode (LED) array when the light passing through the optical system forms an LED plane, in which the initial material polymerization occurs. These methods are characterized by high fidelity and the absence of high-temperature heating of the material. As for DLP, it provides a high print speed due to a simultaneous solidification of the whole layer. Works [11,12,13,14] present the first positive results in the development and investigation of photocurable binding agents and HEM-based highly filled slurries for SLA 3D printing. Some of the research shows the first positive result of using standards for DLP 3D printing materials as a binder for HEM composition, such as 2-hydroxyethyl acrylate (HEA) and polyethylene glycol diacrylate (PEGDA). For example, the authors of [11] have shown promising results in the development of the initial photocurable HEM composition consisting of 50 wt.% RDX, 25 wt.% acrylate binder and 25 wt.% energetic plasticizer. However, the binder composition could be further tailored to improve the filler content, keeping the appropriate mechanical properties and improving energetic characteristics. Improving energetic characteristics following the research [12,14] is also possible by modifying typical oligomers with some active groups in order to make the binder composition active, which will provide the possibility of decreasing the filler while keeping the high energetic characteristics of the material.

Therefore, a UV photocurable binding agent, which allows the introduction of a HEM and high-fidelity 3D printing of products possessing with the best combination of physical, mechanical and energetic properties remains a relevant choice.

In this regard, this work focuses on the UV photopolymerization, digital light processing, and physical, mechanical, and energetic properties of the UV-photopolymerized binder used in stereolithography and filled with high-energy powder. Novel research results presented herein provide additional knowledge about 3D printing of high-energy materials and can be used in the development of more complex compositions and further improvement of DLP 3D printing.

## 2. Materials and Methods

### 2.1. Development and Investigation of Photocurable Slurry

High-molecular compounds, such as epoxy, carbamide, PF resins and natural, urethane, butadiene, styrene–butadiene, isobutadiene, and thiokol rubbers, are conventionally used as a matrix or binder in high-energy systems. Such a matrix binds all components into a mass and provides it with desired mechanical properties (strength, elasticity). These matrices are divided into inert (without oxidizing elements O, Cl, F) and active (with oxidizing elements). In this work, the choice of the polymer material is determined by the presence of oxidizing elements, i.e., the polymer matrix must be active. In this work, we use 70 wt.% urethane dimethacrylate and 30 wt.% 2-hydroxyl methacrylate as UV photocurable binder. Diphenylphosphine oxide (2,4,6-trimethylbenzoyl) in the amount of 3% of the acrylate content is used for photoinitiation. This composition is commercially available and produced by HARZ Labs (Russia) for DLP 3D printing.

Ammonium salts with particle sizes ~50 µm and 165–315 µm were used as an inorganic oxidizer. These particles were preliminary mixed in the ratio of 2 to 3, respectively. For comparison, a monodisperse oxidizer with particle sizes of ~50 µm was also used to prepare photocurable slurry. The obtained powder oxidizer was gradually added to the UV photocurable resin at constant mixing until a slurry with 70 wt.% powder was obtained. The weight content of the oxidizer was determined by the calculated weight content of the components. In calculations, it was assumed that the composition was a mechanical mixture of individual substances, in which the photopolymer composition had the chemical formula C_7_H_13_NO_4_. Calculations were conducted to obtain the excess oxidizer ratio α = 0.5–1, keeping the appropriate viscosity of the slurry [15,16].

At first, we determined the main parameters of the slurry polymerization. For that, we empirically measured the monolayer thickness of the material after UV curing on a 405 nm wavelength, 6.5 mW/cm^2^ power, and 3 to 65 s exposure time. Based on the experimental data, polymerization parameters were determined by using the Beer–Lambert law. The latter describes the curing depth *C_d_* by the following equation [17,18,19,20]:

(1) $$C_{d}=D_{p}\ln(\frac{E_{0}}{E_{c}}),$$

where *E*_0_ is the light energy, *E_c_* is the critical energy or the minimum energy required for resin solidification, and *D_p_* is the penetration depth of curing light. *E_c_* and *D_p_* are constant values of the UV-curable slurry. The relation *C_d_*-*E*_0_ plotted in semilog coordinates allows calculating the *D_p_* parameter from the equation of approximating line. The critical energy *E_c_* can also be calculated from (1). Then, it is possible to determine 3D printing parameters, such that the monolayer thickness is lower than the curing depth, which provides good adhesion between layers.

Besides UV curing parameters, overcuring was studied for the proposed UV photocurable slurry depending on the time of the UV radiation exposure. Overcuring is the key parameter that determines the fidelity of the required three-dimensional geometry of product. Overcuring of the proposed UV photocurable slurry was determined analogously to the method of measuring the slurry curing depth. During this measurement, the control for dimensions was performed along *x* and *y* axes of the polymerized region relative to the specified geometry of the region exposed to the UV radiation.

### 2.2. 3D Printing

**Figure 1:**
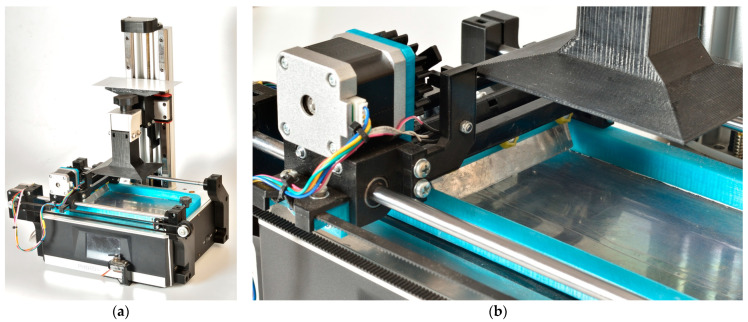
Photographs of the DLP system: (**a**) Full view. (**b**) Doctor blade moving system.

A Photon Mono X 3D printer (Anycubic, Shenzhen, China) was used for digital light processing. Since the obtained UV photocurable slurry was characterized by high viscosity, limiting its effective uniform distribution over the print area, the Photon Mono X was additionally equipped with automatic doctor blade, as shown in Figure 1.

The doctor blade provided an automatic uniform distribution of the material in the overcuring region prior to printing each layer. After printing, the obtained samples were washed in isopropyl alcohol to remove unpolymerized residual material.

### 2.3. Characterization

Scanning electron microscopy (SEM) was carried out on a MIRA microscope (Tescan, Czech Republic, Brno) to investigate the fine structure of the printed samples. The density was measured by hydrostatic weighting in alcohol. The test machine INSTRON 3369 (Instron, Norwood, MA, USA) was used to measure compressive strength and ultimate tensile strength (UTS) of the samples. The compressive strength measurements were performed with the crosshead speed of 0.3 mm/min, and the UTS tests were performed with the crosshead speed of 50 mm/min. The thermogravimetric analysis (TGA) and differential scanning calorimetry (DSC) were carried out on a thermal analyzer Netzsch STA 442 (Netzsch, Zelb, Bavaria, Germany). The samples weighing 2 mg each were manipulated in ceramic crucible in a holder at a heating rate of 10 K/min in inert argon gas and temperature range of 25 °C to 550 °C. Preliminary studies are concerned with the burning process of the samples. It was conducted in a constant volume bomb at a pressure ranging from 0.1 to 4 MPa and recorded with a high-speed camera. The bomb was a 3 L closed steel vessel. Windows on its opposite sides are intended for observation and photo and video recording of the burning process.

## 3. Results

### 3.1. Polymerization Parameters of the Photocurable Slurry

**Figure 2:**
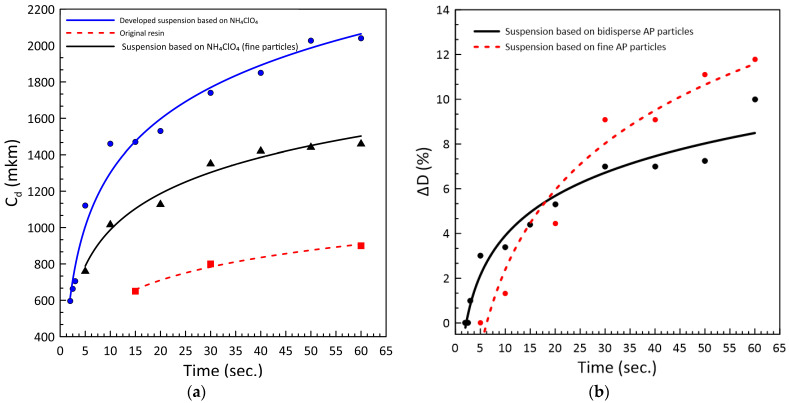
(**a**) Curing depth of the developed suspension and the initial resin. (**b**) Dimensional deviation (overcuring) of the developed suspension, depending on the exposure time.

In Figure 2, experimental curves are provided for the polymerization parameters of the proposed UV photocurable slurries based on ammonium perchlorate (AP) and the initial binder.

In Figure 2, time dependences of the curing depth and the overcuring area behave logarithmically. With increasing exposure time from 5 to 10 s, both parameters drastically grow, while with its successive increase, their growth becomes gradual. The powder addition significantly increases the monolayer thickness of the slurry relative to that of the initial resin at the same exposure time (Figure 2a). Thus, the thickness of the obtained UV photocurable slurries varies between 600 µm and 1.5–2 mm, while the thickness of the monolayer formed after polymerization of the initial binder is not over 1 mm. According to Figure 2b, overcuring does not exceed 2% at the exposure time of 5 s, which is sufficient for the 600 µm thick layer to be polymerized. Overcuring increases by up to 10% with further increasing time of exposure.

**Figure 3:**
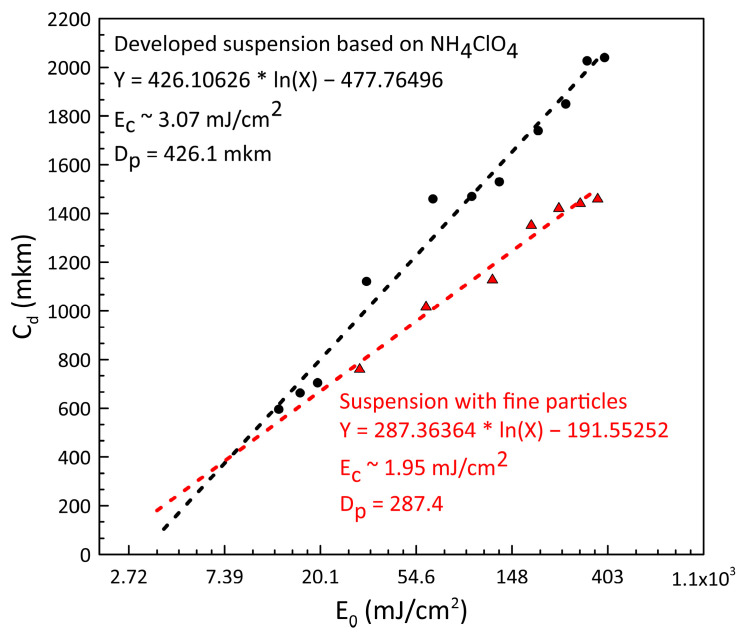
The dependence of curing depth on the exposed UV light dose.

Figure 3 presents the curing depth energy dependence in semilog coordinates. From the logarithmic curve equation, we calculate the penetration depth of the UV light for the developed slurries: for the bidisperse AP-based slurry *D_p_* = 426.1 µm and for the fine-grained AP-based slurry *D_p_* = 287.4 µm. Having solved this equation, we obtain *E_c_* = 3.07 mJ/cm^2^ for the slurry filled with bidisperse AP and *E_c_* = 1.95 mJ/cm^2^ for the slurry filled with fine-grained AP powder, which matches 0.47 s and 0.3 s of exposure to obtain solid layers with the minimum thickness, respectively.

Based on the results, we obtained 3D printing parameters for the slurry with 70 wt.% AP. The first layer was cured for 60 s for the best adhesion to the 3D printing platform. Successive layers were cured for 30 s for their polymerization. The overall printing parameters are presented in Table 1.

### 3.2. 3D Printing and Investigation of HEMs

**Figure 4:**
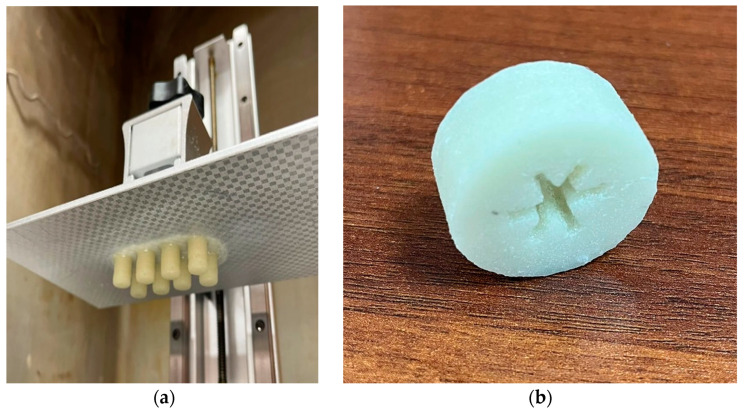
(**a**) Photograph of the 3D-printed samples. (**b**) Sample with developed inner surface.

**Figure 5:**
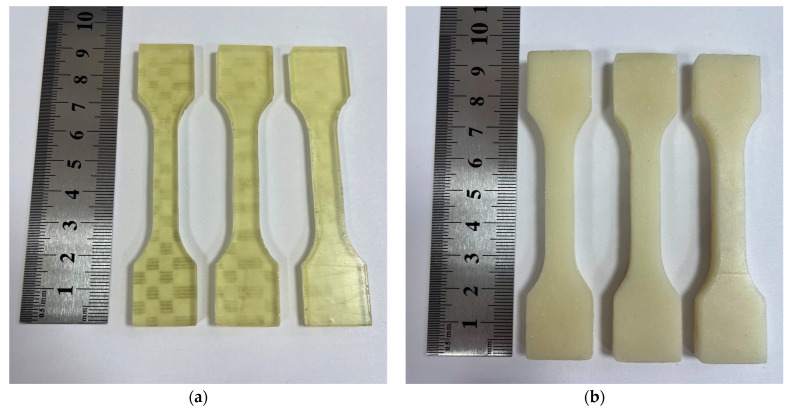
Photograph of the 3D-printed samples for the UTS tests: (**a**) Samples from the initial resin. (**b**) Samples from the bidisperse AP-filled slurry.

Cylinders with a diameter of ~10 mm and ~14 mm height were printed for experiments using the bidisperse AP-filled slurry, as presented in Figure 4a. For the UTS tests, samples with an average working zone of 30 mm length were printed from both the initial resin and the bidisperse AP-filled slurry (Figure 5). Additionally, a cylinder with a developed inner surface, as shown in Figure 4b, was printed to evaluate the possibility of generating the structure with the specified complex geometry.

According to hydrostatic weighting, the density of the obtained samples is 1.475 g/cm^3^. Thus, the porosity is 8%, as the calculated theoretical density of the photopolymer filled with the 70 wt.% AP powder is 1.6 g/cm^3^.

**Figure 6:**
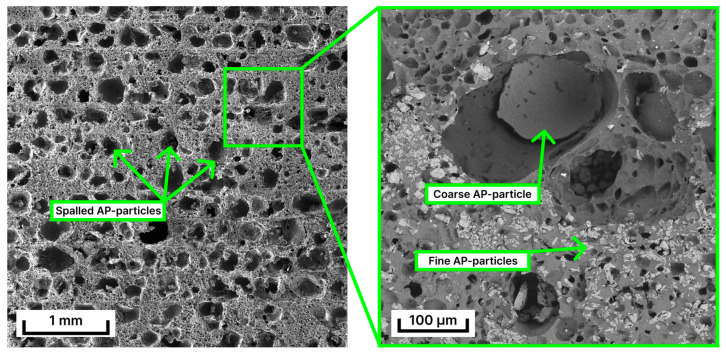
Cross-sectional SEM images of 3D-printed sample microstructure.

Cross-sectional SEM images in Figure 6 show the microstructure of the printed sample. One can clearly see the layer structure of the fabricated material with a layer size of 250 to 300 µm. The inner structure consists of many micro- and macropores. The size of the latter is 300 µm, similar to the coarse AP particle size. The micropore size ranges from several microns to ~50 µm, which is comparable to that of fine AP particles.

**Figure 7:**
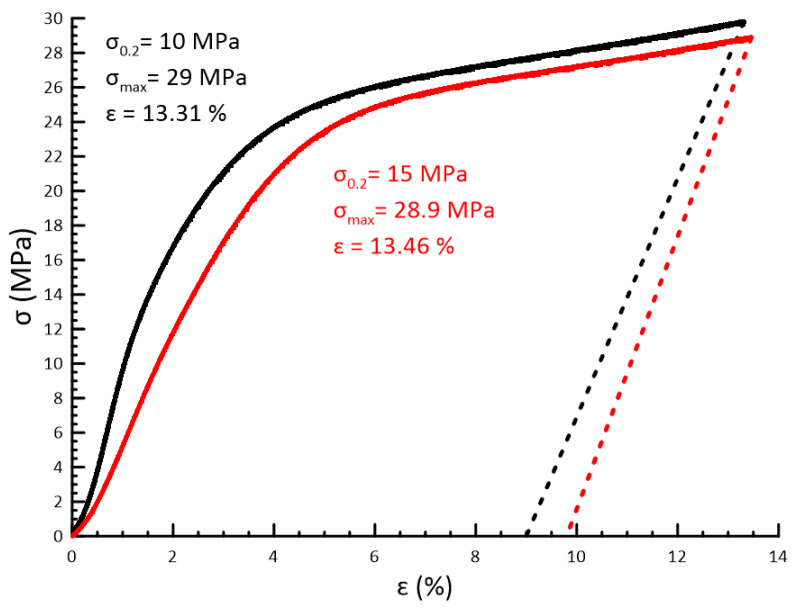
Compression test stress–strain curves of 3D-printed samples (black and red lines–sam-ples No. 1 and No. 2 respectively; dotted lines—relaxation after compression test).

Figure 7 presents stress–strain curves of fabricated samples. According to this figure, the elastic strain is ~3%, while the yield stress ranges between 10 and 15 MPa. Observed plastic deformation does not lead to material fracture and defect formation.

Samples are loaded until ~13% deformation. After successive loading, the stress–strain curve demonstrates a gentle slope after reaching the yield strength. The maximum stress is 29 ± 3 MPa. It is found that elastic stress relaxation on the order of 3% occurs after unloading.

**Figure 8:**
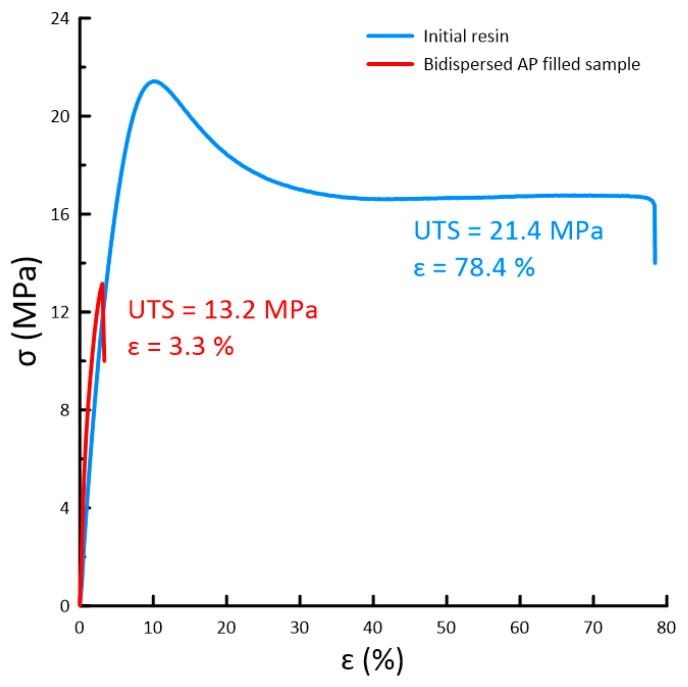
UTS test stress–strain curves of 3D-printed samples.

The results of the UTS tests are presented in Figure 8.

According to the result of the tensile strength test, the UTS of the initial resin is 22 ± 2.2 MPa with maximum elongation up to 80%. When adding the bidisperse AP powder filler, the UTS decreases to 13 ± 1.3 MPa with a maximum elongation of 3%.

### 3.3. Thermogravimetric Analysis and Burning Rates

**Figure 9:**
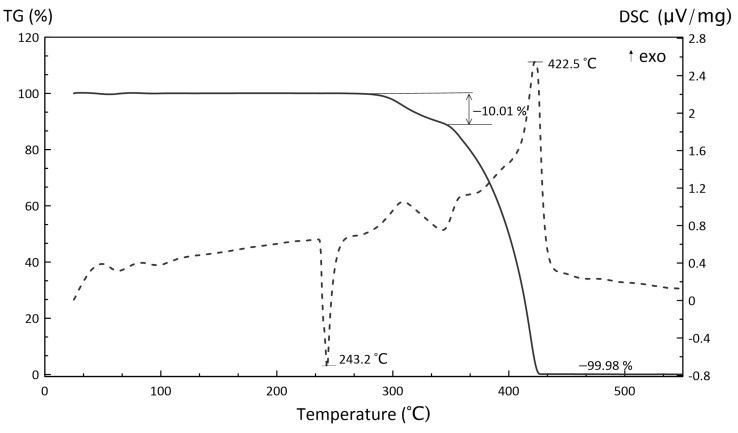
TGA (solid line) and DSC (dotted line) curves of AP decomposition.

**Figure 10:**
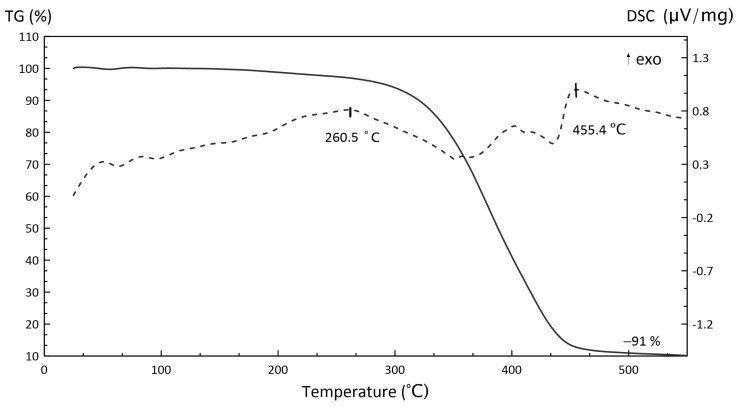
TGA (solid line) and DSC (dotted line) curves of photopolymer decomposition.

**Figure 11:**
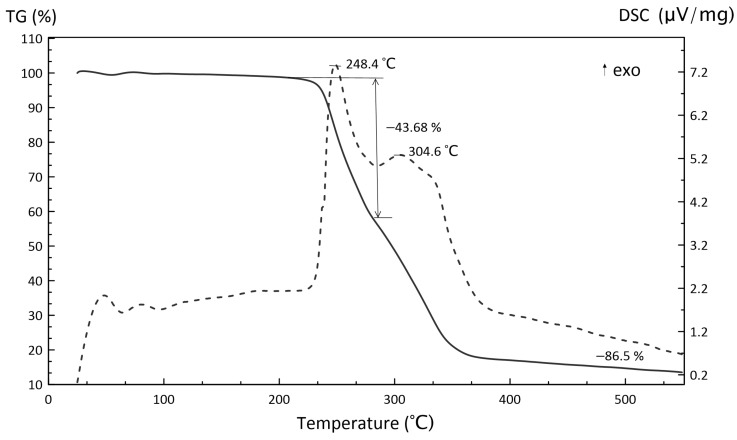
TGA (solid line) and DSC (dotted line) curves of AP–photopolymer system decomposition.

Figure 9, Figure 10 and Figure 11 illustrate the TGA results of several components (inorganic oxidizer and photocurable resin) and the obtained slurry.

According to the TGA curve, a complete ammonium perchlorate decomposition is observed at 549 °C by the autocatalytic mechanism. Its residual mass is 0.02% of the initial weight. We can assume either that the endothermal peak at 243.2 °C correlates to the AP melting or that its rhombic crystal structure transfers to a cubic. The weight loss begins between 309.2 and 342.5 °C when the AP decomposition is accompanied by the release of chlorine gas, oxygen, and vapor. The final AP decomposition is observed at the second endothermal peak at 422.5 °C. The TGA curve shows the complete decomposition of the oxidizer, its residual mass being 0.02% of the initial weight.

According to the TGA curve shown in Figure 10, the UV photocurable resin decomposition is almost complete; at 549 °C, its residual mass is ~10% of the initial. The endothermal region up to 379.2 °C correlates with melting, after which the endothermic reaction is accompanied by radical formation and removal from the sample surface.

When considering the AP–polymer system decomposition presented in Figure 11, it should be noted that its residual mass does not change as compared to the initial binder and probably consists of polymer decomposition products. In this system, a high exothermal peak is observed at 248.4 °C, which nucleates at 238.5 °C, while the second peak is observed at 304.6 °C. This indicates the shift of the reaction initiation toward low temperatures.

**Figure 12:**
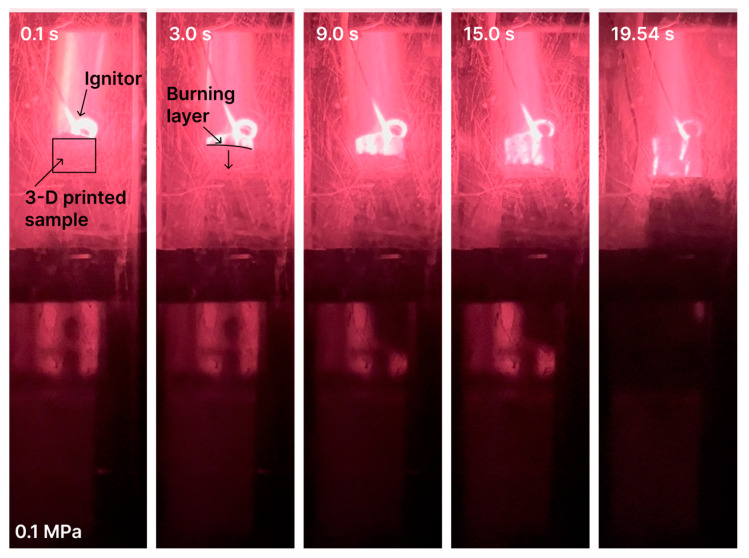
Burning of 3D-printed AP–photopolymer sample under atmospheric pressure.

**Figure 13:**
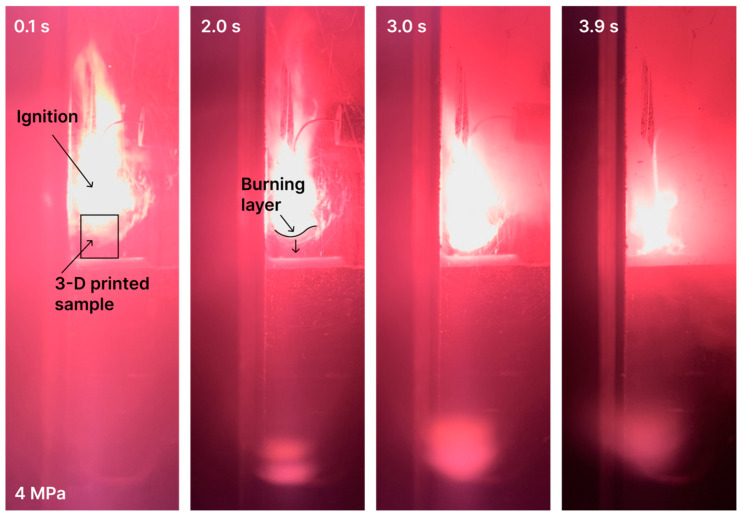
Burning of 3D-printed AP–photopolymer sample under 4MPa pressure.

Video footage in Figure 12 and Figure 13 demonstrates the burning process under the atmospheric and 4 MPa pressures. One can see that the burning process is stable, layer-by-layer, and has no failure or extinction.

**Figure 14:**
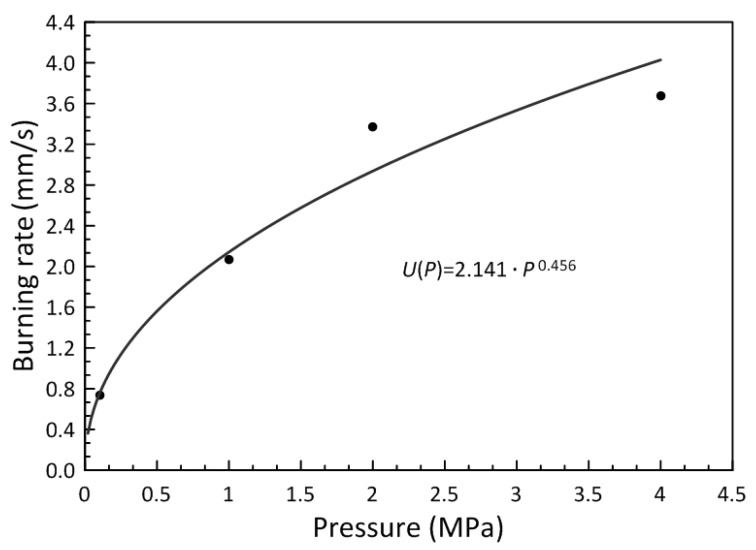
Burning rate–pressure dependence.

Figure 14 presents the plot of the burning rate and pressure relation for AM samples.

The obtained data are well approximated by the exponential function, which correlates with the power law of burning (Vieille relation) [21]

(2) $$u(p)=u_{0}aP^{v},$$

where *p* is the pressure, *a* is the empiric constant, and *ν* is the exponent quantity in the burning rate law, which describes the sensitivity of the burning rate to the pressure changes, i.e., $\nu {=(\frac{\partial \ln u}{\partial \ln p})}_{T}$. According to the obtained equation of the approximated curve, the exponent quantity ranges from 0.3 to 0.5, which indicates a low sensitivity of the burning rate to the pressure changes.

## 4. Discussion

**Figure 15:**
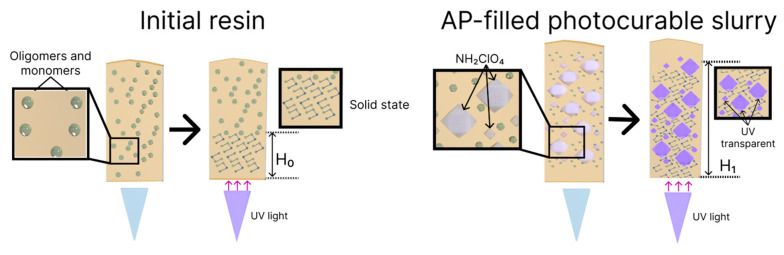
Proposed mechanism of UV polymerization of the slurry with and without AP particles.

Our studies indicate that the proposed UV photocurable material can be used as a binder to create high-energy compositions for DLP 3D printing. The thickness growth of the curing monolayer after the addition of the powder oxidizer to the initial UV photocurable resin can be associated with the UV transparency of AP particles [21]. Coarse (300 µm) particles can, therefore, scatter the UV radiation deep into the curable slurry, thereby increasing the thickness of the curable layer. The proposed mechanism of the UV polymerization of the slurry highly filled with AP particles is illustrated in Figure 15.

We can thus conclude that the growing UV penetration depth of the slurry compared to the initial resin cured at the same energy dose could, on the one hand, increase the printing rate and, on the other hand, exert a negative effect on the fidelity and induce overcuring (see Figure 2). The results of the curing depth test (Figure 2 and Figure 3) prove this theory. Thus, for the slurry filled with fine-grained AP powder (~50 µm), the curing depth decreases at the same exposure time and power compared to bidisperse AP powder filler slurry.

The DLP samples possessed a heterogeneous lamellar structure with a uniform distribution of AP particles without agglomeration. The obtained microstructure included large cavities ranging in size from 200 to 300 µm and 50 µm pores. The porosity could be attributed to the low particle adhesion to the binder, which spall out of the outer material surface. Similar results were obtained by other authors [11] but for the RDX particles in a photopolymerized matrix. SEM images in Figure 6 confirmed the similarity of AP particles with the morphology and size of observed pores. In the meantime, fine AP particles (≤50 µm), according to Figure 6, are less subjected to spalling. That indicated the appropriateness of using only fine (20–40 µm) AP particles as a filler. There is, however, no information in the literature about the resulting effect of such particles on the thermal decomposition parameters of the obtained HEM that can be investigated in further research.

Elastic properties of the produced material and its compressive strength, up to 10–15 MPa without plastic deformation and up to 29 ± 3 MPa without fracture, were stipulated by the elastic behavior of the loaded initial binder based on urethane methacrylate. The obtained UTS test results show good elasticity of the initial resin with the UTS of 22 ± 2.2 MPa and maximum elongation up to 80%. When adding the bidisperse AP powder filler, the UTS decreases to 13 ± 1.3 MPa with a maximum elongation of 3%. Thus, the AP-filled polymer is significantly more brittle compared to the original resin when tensile stresses are applied. However, compared with the literature data, the maximum compressive strength obtained by the authors [11] reaches 19.6 MPa for the material consisting of 2-hydroxyethyl acrylate (HEA) and polyethylene glycol diacrylate (PEGDA). Another group of researchers [22] presents the results of an extrusion-based 3D printing approach following UV curing to obtain HEM structures. The UTS of the obtained materials with the binder based on HTPB and photocurable composition reported by the authors [22] reaches not more than 1 MPa. However, the reported elongation is higher compared with current research and reaches up to 15%.

The obtained mechanical properties allowed the obtaining of the appropriate mechanical characteristics of HEM-based products fabricated by DLP 3D printing with the use of the proposed UV photocurable material.

According to TGA/DSC curves, the obtained HEM possessed high exothermicity. Importantly, the thermal analysis was conducted in argon without additional oxidation. It was, therefore, assumed that the high exothermicity was conditioned by the oxidizer–polymer interaction and/or their gaseous phase. The TGA/DSC curves for polymers and polymer composites were difficult to investigate owing to peak overlaps provided by simultaneously occurring processes. Therefore, it is advisable to use calorimetric techniques to study heat generation processes more thoroughly.

The burning process of DLP-produced three-dimensional samples, based on the UV photocurable material, was uniform and stable, without failure or extinction. At the pressure growth from 0.1 to 4 MPa, the burning rate increased from 0.74 to 3.68 mm/s, respectively.

All these results describing polymerization, structure, mechanical properties, and thermal decomposition of the HEM can be used in DLP 3D printing, and the proposed UV photocurable material can be utilized in the fabrication of HEM-based parts with complex geometry and specified burning and energy parameters.

## 5. Conclusions

(1) The obtained AP-filled (70 wt.%) UV-curable urethane dimethacrylate-based slurry is suitable for DLP 3D printing of HEM structures;
(2) The curing parameters of the obtained slurry significantly depend on the AP powder dispersity. Thus, for the bidisperse AP-based slurry, *D_p_* = 426.1 µm, and for the fine-grained (~50 µm) AP-based slurry, *D_p_* = 287.4 µm. The curing depth of the obtained slurries increases compared to the initial resin due to the transparency of the AP particles to the UV light;
(3) The obtained 3D-printed samples filled with bidisperse AP powder withstand compressive strength up to 29 ± 3 MPa and tensile strength up to 13 ± 1.3 MPa. The compressive deformation without fracture reaches up to 13%, while maximum tensile elongation reaches only 3%;
(4) According to TGA/DSC curves, the obtained HEM possessed high exothermicity with the shift of the start of the thermal decomposition reactions of the slurry to the lower temperatures compared to the initial AP powder and photocurable resin separately;
(5) The burning process of 3D-printed samples filled with bidisperse AP powder was uniform and stable, without failure or extinction. At the pressure growth from 0.1 to 4 MPa, the burning rate increased from 0.74 to 3.68 mm/s, respectively.

## Figures and Tables

**Table 1:** Printing parameters.

Parameter	Base Layer	Other Layers
UV source power, mW/cm^2^	6.5	6.5
Curing time, s	60	30
Layer thickness, µm	250	250

## Data Availability

The data presented in this study are available in the article.
